# Multiple Resistances and Complex Mechanisms of *Anopheles sinensis* Mosquito: A Major Obstacle to Mosquito-Borne Diseases Control and Elimination in China

**DOI:** 10.1371/journal.pntd.0002889

**Published:** 2014-05-22

**Authors:** Xuelian Chang, Daibin Zhong, Qiang Fang, Joshua Hartsel, Guofa Zhou, Linna Shi, Fujin Fang, Changliang Zhu, Guiyun Yan

**Affiliations:** 1 Department of Pathogen Biology, Nanjing Medical University, Nanjing, China; 2 Department of Pathogen Biology, Bengbu Medical College, Anhui, China; 3 Program in Public Health, College of Health Sciences, University of California at Irvine, Irvine, California, United States of America; Technical University of Mombasa, Kenya

## Abstract

Malaria, dengue fever, and filariasis are three of the most common mosquito-borne diseases worldwide. Malaria and lymphatic filariasis can occur as concomitant human infections while also sharing common mosquito vectors. The overall prevalence and health significance of malaria and filariasis have made them top priorities for global elimination and control programmes. Pyrethroid resistance in anopheline mosquito vectors represents a highly significant problem to malaria control worldwide. Several methods have been proposed to mitigate insecticide resistance, including rotational use of insecticides with different modes of action. *Anopheles sinensis*, an important malaria and filariasis vector in Southeast Asia, represents an interesting mosquito species for examining the consequences of long-term insecticide rotation use on resistance. We examined insecticide resistance in two *An. Sinensis* populations from central and southern China against pyrethroids, organochlorines, organophosphates, and carbamates, which are the major classes of insecticides recommended for indoor residual spray. We found that the mosquito populations were highly resistant to the four classes of insecticides. High frequency of *kdr* mutation was revealed in the central population, whereas no *kdr* mutation was detected in the southern population. The frequency of G119S mutation in the *ace-1* gene was moderate in both populations. The classification and regression trees (CART) statistical analysis found that metabolic detoxification was the most important resistance mechanism, whereas target site insensitivity of L1014 *kdr* mutation played a less important role. Our results indicate that metabolic detoxification was the dominant mechanism of resistance compared to target site insensitivity, and suggests that long-term rotational use of various insecticides has led *An. sinensis* to evolve a high insecticide resistance. This study highlights the complex network of mechanisms conferring multiple resistances to chemical insecticides in mosquito vectors and it has important implication for designing and implementing vector resistance management strategies.

## Author Summary

Malaria and lymphatic filariasis are two of the most important mosquito-borne parasitic diseases worldwide, which can occur as concomitant human infections. The *Anopheles sinensis* mosquito is the most common malaria and lymphatic filariasis vector in Southeast Asia. Disease-vector control is an important part of the global malaria and filariasis control strategies. Pyrethroid insecticides are the major vector control agents, and rotational or combinational use with other classes of insecticides has been proposed to mitigate the problem of pyrethroid resistance. Therefore, assessing resistance to multiple classes of insecticides is important because vector control programs may not be effective, or even fail, if mosquito vectors are already resistant to the insecticides being used in rotation or combination. The field populations of *An. sinensis* mosquito from central China (Anhui) and southern China (Yunnan) developed high resistance to four classes of insecticides tested, including pyrethroids (deltamethrin and permethrin), organochlorine (DDT), organophosphate (malathion), and carbamate (bendiocarb). This study examined two questions: 1) the consequences of long-term insecticide rotation use on insecticide resistance, and 2) the role of metabolic detoxification and target site insensitivity in resistance to chemical insecticides. The information obtained from this investigation is valuable in informing current and future vector control strategies.

## Introduction

Malaria and filariasis are two of the most important vector-borne parasitic diseases in Southeast Asia. Although China and several other countries in the region have reported a marked downward trend in malaria cases, high malaria incidence has been observed in the major neighboring endemic countries such as Myanmar, Bangladesh, and India [1]. Cross-border migration provides increased opportunities for malaria infections. Further, the high frequencies of natural disasters augment the risk of imminent outbreaks of malaria. Therefore, malaria surveillance and vector control become very important tools to prevent malaria outbreaks in low-transmission areas [1]. Currently, insecticide-treated bed nets (ITNs) and indoor residual spray (IRS) are the primary vector control tools in the Global Strategy for Malaria Control and the Roll Back Malaria program [2] and in the Global Fund to Fight AIDS, Tuberculosis and Malaria [3]. Pyrethroids are currently the only class of insecticide approved for use on ITNs [4] due to their high toxicity to insects, rapid rate of knockdown, strong mosquito excito-repellency, and low mammalian toxicity [5]. Reducing vector-human contact by the use of ITNs has been shown effective in reducing malaria transmission [6], [7]. However, the emergence and spread of insecticide resistance has significantly hampered the efficacy of ITN programs [8]. Insecticides remain the most important vector control method; however, insecticide resistance poses a major threat to vector-borne disease control due to lack of other viable alternatives.

Several methods have been proposed to mitigate insecticide resistance in vector mosquito populations, including insecticide rotation strategies [1] and combinational use of insecticides with different modes of action. Resistance to any particular insecticide is mitigated, since the selection pressure is removed before resistance is developed. Combinational use of insecticides with different modes of action is currently applicable to IRS only, with the assumption that mosquito vectors exhibit low resistance to the new insecticides under consideration. Adding IRS to the ITN program becomes an increasingly popular malaria control strategy worldwide due to increasing resistance to pyrethroids used in ITNs [8]. WHO-recommended products for IRS include four classes of insecticides: pyrethroids, organochlorines, organophosphates, and carbamates [1]. However, the long-term consequences of insecticide rotation and combinational use of insecticides on mosquito insecticide resistance are not clear.

The mosquito *Anopheles sinensis* is the most important malaria vector in China and other Southeast Asian countries [9]–[14]. In southern China, *An. sinensis* play an important role in the natural transmission of both malaria and filariasis (*Wuchereria bancrofti*) [15]–[17], as well as *Romanomermis jingdeensis* [18] and *Setaria digitata* [19]–[21]. The major breeding sites of *An. sinensis* in China are rice fields where various classes of insecticides have been used in agricultural pest control regimes in rotation [22], [23]. Although *An. sinensis* is not the intended target in this pest control regimes, it has been directly exposed to the insecticides over a period of four decades [24]–[26]. Therefore, *An. sinensis* in China also represents an interesting model in which to examine the consequence of long-term rotational use of various insecticides on resistance evolution. In this study, we examined the extent and distribution of insecticide resistance in *An. sinensis* against the four classes of insecticides recommended by WHO for malaria vector control by IRS. It also indirectly examined the long-term consequences of rotational use of insecticides on mosquito resistance evolution. Using *An. Sinensis* populations from central and southern China, general correlations between insecticide-specific resistance and geography can be examined. The information obtained from this investigation can be used to guide insecticide rotation strategies.

Another major objective of this study was to examine the importance of target site insensitivity and various metabolic detoxification enzymes in resistance to the major classes of insecticides used in IRS. Pyrethroids and organochlorines function as neurotoxins that act by prolonging sodium channel activation whereas organophosphates and carbamates kill insects by inhibiting acetylcholinesterase found in the central nervous system [27]. The voltage-sensitive sodium channel proteins are the major target site for pyrethroids and DDT, and a mutation at codon 1014 the *para* sodium ion channel gene causes knockdown resistance (*kdr*) [27]. On the other hand, a mutation at codon 119 of the acetylcholinesterase (*ace-1*) gene that leads to a single amino acid substitution of glycine to serine in the binding pocket of acetylcholinesterase may confer resistance to organophosphates and carbamates. In addition to target-site insensitivity, metabolic detoxification enzymes—including cytochrome P450 monooxygenases (P450s), carboxylesterases, and glutathione S-transferases (GSTs) —may also augment insecticide resistance [27]. The possible pleiotropic role of metabolic enzymes on resistance and the relative significance of each mechanism on resistance to multiple classes of insecticides in *An. sinensis* are unknown. This information is particularly valuable to the development of reliable molecular-based resistance surveillance tools.

## Materials and Methods

### Ethics statement

No specific permits were required for the described field studies. For mosquito collection in rice paddies, oral consent was obtained from field owners in each location. No sites were protected by law and this study did not involve endangered or protected species.

### Study sites

**Figure 1:**
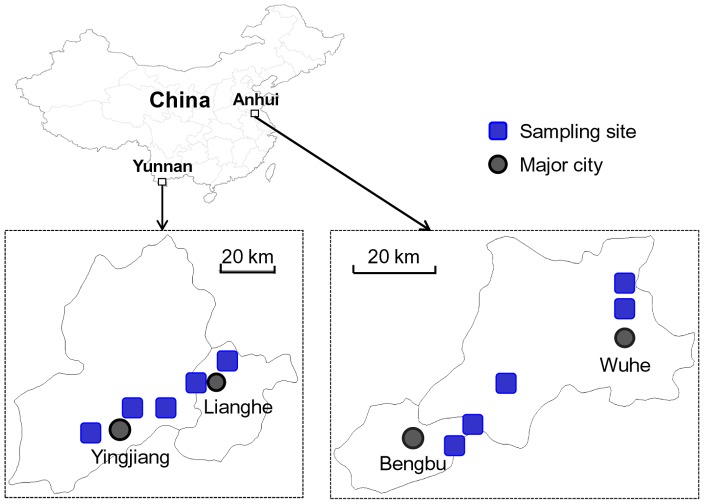
Sampling sites of *Anopheles sinensis* mosquitoes in southern (Yunnan) and central (Anhui) China.

The study was conducted in malaria endemic sites in southern China (Yunnan Province) and central China (Anhui Province) (Fig. 1). Yunnan Province has the highest malaria incidence in China and is responsible for about 50% of officially reported malaria cases in China [28]. Malaria in Yunnan province is mesoendemic with perennial circulation of both *P. vivax* and *P. falciparum* parasites [28], [29]. Anhui Province is hypo-endemic, with *Plasmodium vivax* as the predominant malaria species [30]. The Yunnan site was located in Yingjiang and Lianghe Counties, Dehong Prefecture, and the Anhui site was in suburbs of Bengbu City. Rice is the major agricultural crop in these study sites, with 1–2 harvests per year. Due to severe insect pest damage to the rice, insecticide use for pest control has been very intensive, with several rounds of sprays administered during each growing season. From 1960 to 1990, insecticides were extensively used in agriculture in China because the government routinely subsidized pesticide expenses by as much as 85%. The pesticides used were predominantly organochlorines and organophosphates from the 1970s up to the early 1980s. Since the mid-1980s, pyrethroids have been the dominant insecticides with pyrethroids-treated areas constituting more than one third of the total insecticide-treated area in China [26], [31]. In addition to their agricultural use, pyrethroids have had various public-health applications—as indoor sprays or incense, impregnated in bed nets, or as tools in public sanitation. Other insecticides, including organophosphates and carbamates, have been used widely but less extensively than pyrethroids.

### Mosquito sample collection

During May–August 2012, *Anopheles sinensis* mosquito larvae and pupae were collected from irrigated rice fields and small ponds with aquatic plants, using standard 350-ml dippers. We used adults reared from field-collected larvae for this study to minimize the influence of mosquito age and blood feeding history on resistance measurements [4], [32]. For each site, we collected mosquito larvae from >100 breeding sites in each of the five villages, separated by 5–10 km from each other, to avoid using genetically-related siblings in the subsequent resistance analysis. We collected a total of 4,000 anopheline larvae per site. The collected mosquito larvae were transported to the local rearing facility to be reared into adults. All adult mosquitoes were identified to species using the published morphological keys of Dong [33]. *An. sinensis* adult mosquitoes were provided with fresh 10% sucrose solution daily.

### Insecticide susceptibility bioassay

After the mosquitoes were identified to species, *An. sinensis* female adult mosquitoes at 3–4 days post emergence were tested for susceptibility to five insecticides belonging to four classes (0.05% deltamethrin, 0.75% permethrin, 5% malathion, 0.1% bendiocarb, and 4% DDT), using the standard WHO resistance tube assay [4]. The discriminating dose used for each insecticide should kill 99.9% susceptible mosquitoes [4]. As a susceptible mosquito control, we used a laboratory susceptible strain that has been maintained in the insectary of the Jiangsu Institute of Parasitic Diseases in Wuxi, China, for more than 10 years with no insecticide exposure. For each insecticide, a total of 100–150 female mosquitoes were tested in insecticide susceptibility bioassays, with 20 mosquitoes per tube. Equal number of mosquitoes were exposed to the corresponding control papers impregnated with silicone oil (deltamethrin/permethrin control), olive oil (malathion/bendiocarb control), and resila oil (DDT control). After a 1-hr exposure, mosquitoes were transferred to recovery cups and maintained on 10% sucrose solution for 24 hrs, and the number of surviving mosquitoes was recorded. Here, we defined resistant for the mosquitoes alive 24 hours after the end of the bioassay and susceptible for the mosquitoes knocked down during the 60 min exposure time or within 24 hr recovery period [34]. Mosquitoes were considered knocked down if they were unable to walk from the center to the border of a 7-cm filter paper disc, either alone or when they were mechanically stimulated [4]. After the resistance/susceptible status were recorded, one leg of each mosquito was removed and preserved individually in 95% alcohol for subsequent DNA analysis, and the remainder of the body was immediately tested for metabolic enzyme activities. Therefore, only fresh mosquitoes were tested for metabolic enzyme activities. Our definition of susceptible mosquitoes was based on knockdown phenotype, rather than death phenotype. As such, metabolic enzyme activities of susceptible mosquitoes could be measured because fresh mosquitoes were used. This definition allowed us to determine the association between metabolic enzyme activities and resistance with little bias because the resistant and susceptible mosquitoes were exposed to the insecticide in the same manner and our analysis computed the ratio of metabolic enzymes in the resistant mosquitoes to the susceptible mosquitoes. A total of 1,103 female adult mosquitoes were used for bioassay in the study.

### Metabolic enzyme activity assays

Three metabolic enzymes were analyzed: cytochrome P450 monooxygenases (P450s), glutathione S-transferases (GSTs), and carboxylesterases (COEs). We followed our previously published protocol to measure monooxygenase and GST activities [23]. Mean absorbance values for each tested mosquito and enzyme were converted into enzyme activity and standardized based on the total protein amount. Total protein was measured for each mosquito using the method of Bradford [35]. All measurements were done in duplicate. COE activity was measured following the method of Hosokawa and Satoh [36]. Briefly, 900 µl of p-nitrophenyl acetate solution (1 mM) was transferred to 1.5-ml test tubes and incubated at 30°C for 5 min, then 100 µl of mosquito homogenates was added and vortexed for 5 sec. The reaction mixture was transferred to 1.0-ml semimicro cuvettes, and the release of p-nitrophenol was measured using a UV/VIS spectrophotometer at 405 nm for 2 min. Spontaneous hydrolysis was used as the blank. COE activity was calculated as µmol of p-nitrophenol formed per min per mg protein, using the formula (Δabsorbance/min – Δblank/min) ×1.0/16.4×0.05× protein (mg/ml). An absorption coefficient of 16,400 M^−1^·cm^−1^ was used [37]. For each mosquito population and each insecticide, 100 female adult mosquitoes were tested.

### Mosquito DNA extraction and molecular identification of mutation at the target sites

One leg of each mosquito was used for DNA extraction for subsequent PCR-based mosquito species confirmation and mutation detection in *kdr* and *ace-1* genes. DNA extraction was done with the SYBR Green Extract-N-Amp™ Tissue PCR Kit (SIGMA) following the manufacturer's protocol. Extracted DNA was stored at 4°C or used immediately. Molecular identifications of An. sinensis species were done by using species-specific primers and amplification of the ITS2 and 28S rDNA regions (D1 and D2) [38]. A total of 100 mosquitoes per site were randomly selected and tested molecularly, and all of them identified as An. sinensis. Detection of point mutation of the kdr gene at codon 1014 was done by using the allele-specific PCR (AS-PCR) methods developed by Zhong et al [23]. A PCR-RFLP method was developed to rapidly determine point mutation of the ace-1 gene at codon 119 following the method used in An. gambiae [39]. Briefly, we designed a pair of primers (Forward primer 467F: GTGCGACCATGTGGAACC, Reverse primer 660R: ACCACGATCACGTTCTCCTC) based on the An. gambiae ace-1 gene sequence (GenBank accession: BN000066) to amplify a 193-bp fragment that flanks the target codon position 119 in the ace-1 gene (ace-1R). The PCR products of 20 individuals from each population were sequenced in both forward and reverse directions (GenBank accession: KF697669- KF697683, KF709027-KF709034). The PCR product was digested by AluI restriction enzyme, which results in 118-bp and 75-bp fragments when there is a homozygous G119S mutation. A total of 577 and 414 mosquitoes were tested for kdr and ace-1 mutations, respectively. Genotype frequencies were calculated and deviation from Hardy-Weinberg equilibrium (HWE) was analyzed using the web-based program ‘GENEPOP’ [40].

### Insecticide use and sales survey

Agricultural activity is particularly intense in the two study sites. Insecticides are commonly used for agricultural pest control. An insecticide usage survey was conducted following a standardized questionnaire that included questions on crops harvested and insecticides used, including brand name, time and operation dosage for crop treatment and for human health protection. For each site, questionnaire surveys on 20 households were administered.

### Residual insecticide analysis in water and soil samples

Soil and water samples were collected from four mosquito sampling sites in Anhui Province to determine residual insecticide concentrations for deltamethrin (pyrethroid) and chlorpyrifos (organophosphate) in July 2012. For each sampling site, water samples were collected at four equidistant points in 250-ml aliquots, 5 cm from the water's surface. The aliquots were combined in 1-L amber glass bottles and analyzed in duplicate. Soil samples were extracted as 10-cm plugs at four points in each sampling site. The samples were combined and manually blended until homogeneous. Water and soil samples were chilled (4°C for water and −20°C for soil) until analysis. A negative water and soil samples for controls were collected from an abandoned cornfield that has not been planted or sprayed with insecticides for at least two years. A positive control sample was prepared by adding diluted deltamethrin and chlorpyrifos to the negative water and soil samples at a concentration in ppm. Each water sample was directly extracted in a separatory funnel with methylene chloride. The organic fractions were combined, dried with anhydrous sodium sulfate, and filtered. The solvent was stripped *in vacuo* before a final dilution in hexane for analysis using a GC-MS equipped with an electron impact ion source. Soil samples were prepared by mixing 5.0 g (dry weight) sample with anhydrous sodium sulfate. The sample was extracted with a 1∶1 (v/v) methylene chloride:acetone solution under ultrasonicating conditions. The organic layers were combined and dried with sodium sulfate, and the solvent was evaporated *in vacuo* prior to the addition of activated copper to remove sulfur contamination. The solution was filtered and evaporated to dryness. The residue was taken up in 9∶1 hexane:acetone and eluted through a plug of silica conditioned with 9∶1 hexane:acetone. The sample was eluted and dried, and the residue was reconstituted in 9∶1 hexane:acetone for analysis using a GS-MS equipped with an electron-impact ion source in selective-ion monitoring mode. Sample chemical analysis was conducted by the National Center of Agricultural Standardization and Supervision (Anhui) under the China National Center for Quality Supervision and Testing of Agricultural-Avocation Processed Food.

### Statistical analysis

Mosquito mortality rates after a 24-hr recovery period were calculated for each insecticide. If control mortality was greater than 5% but less than 20%, then the observed mortality was corrected according to the mortality rates of the respective control groups (control paper) using Abbott's formula following the WHO test procedures [41]. If the control mortality was below 5%, it was ignored and no correction was necessary. If the control mortality was above 20%, the tests were discarded. We classified mosquito resistance status according to WHO criteria [4]—i.e., resistant if mortality is <90%, probable resistant if mortality is 90–98%, and susceptible if mortality is >98%. Univariate analysis of variance (ANOVA) was conducted using the *arcsin* transformation of the mosquito mortality rate to determine among-population differences in mosquito mortality rates in the insecticide susceptibility bioassay. One-tailed Mann-Whitney tests were used to compare the enzyme activities in the two field populations and the lab susceptible strains.

To determine the role of target site mutation and metabolic detoxification enzymes on phenotypic resistance, we conducted the following three analyses. First, the *kdr* and *ace-1* allele frequency was calculated in each population. The odds ratio (OR) of *kdr* and *ace-1* gene mutation on resistance (survival or death in resistance bioassay) was calculated, and the statistical significance was determined using the Chi-Square (χ^2^) test. Second, the mean enzymatic activity was calculated for mosquitoes that survived the bioassay (resistant) and those that died in the bioassay (susceptible) for each insecticide tested, and the relative enzyme activity ratio of resistant individuals to susceptible individuals was presented. A t-test was used to determine whether this ratio value was significantly different from the null expectation of 1 (same enzyme activities between resistant and susceptible individuals). Third, we used the CART method to determine the relative contributions of target site mutations (*kdr* and *ace-1* genes) and metabolic detoxification enzymes (P450s, GSTs and COEs) to phenotypic resistance. The CART method is a nonparametric statistical method that recursively partitions the multidimensional space defined by the explanatory factors into subsets as homogeneous as possible [42]. In the CART analysis, the dependent variable was resistant or susceptible status of a mosquito, and factors analyzed were *kdr* or *ace-1* mutation (binomial variables), and P450, GST and COE enzyme activities (continuous variables). We used the Gini impurity criterion to determine variable splits and identified optimal trees from repeated cross-validations to find the smallest trees whose model errors fell within 1 standard error of the minimum error [43]. The relative importance of each variable is measured by the predictor importance score. A raw variable importance score is constructed by locating every node split by a variable and summing up all the improvement scores generated by the variable at those nodes (if the variable acted as a surrogate, add up all those improvement scores as well). The raw importance score is rescaled so that the best score is always 100 and all other variables are scaled down proportionately [44]. The CART v6.0 software (Salford Systems, Inc) was used to construct classification and regression trees [44]–[46].

### Accession numbers

***An. sinensis kdr*** **haplotypes.** Yunnan: KF697669-KF697673; Anhui: KF697674-KF697683.

***An. sinensis ace-1*** **haplotypes.** Yunnan: KF709027-KF709030; Anhui: KF709031-KF709034.

## Results

### Insecticide susceptibility bioassay

**Figure 2:**
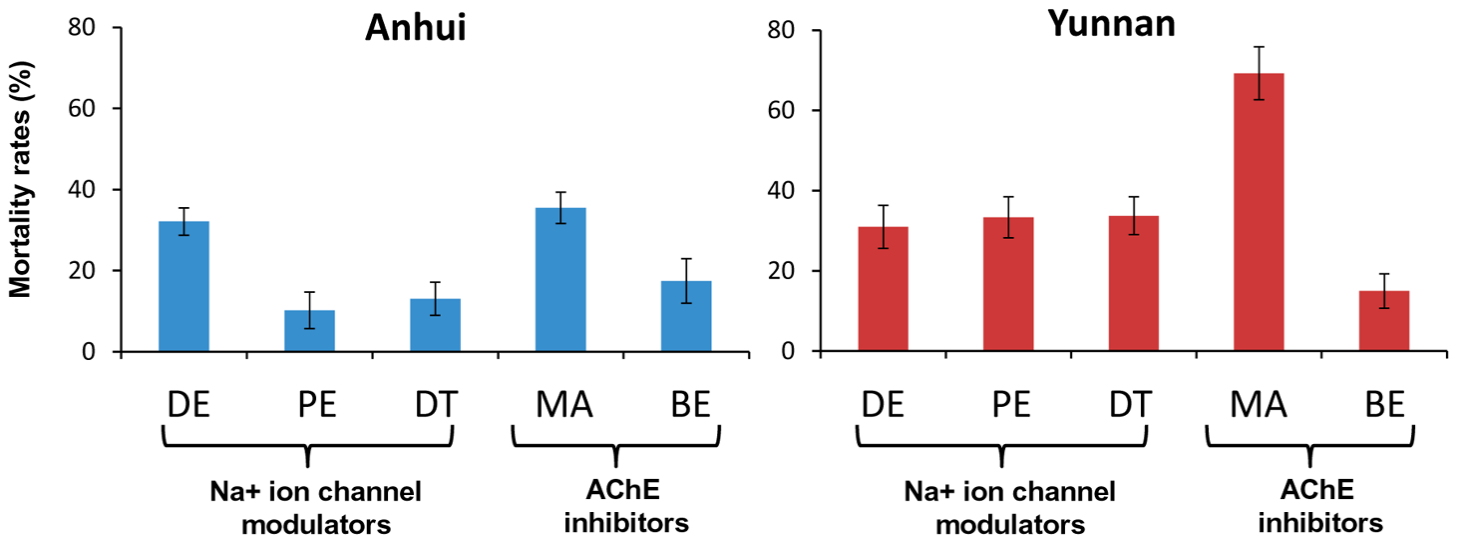
*Anopheles sinensis* mosquito mortality rates to multiple classes of insecticides in the standard WHO tube resistance bioassay. Two mosquito populations (Anhui in central China and Yunnan in southern China) were tested. Insecticides tested and concentrations were: DE (0.05% deltamethrin), PE (0.75% permethrin), DT (4% DDT), MA (5% malathion) and BE (0.1% bendiocarb). Insecticide resistance classification based on 2013 WHO criteria: susceptible if mortality rate >98%, probable resistant if mortality rate ranges 90–98%, resistant if mortality rate <90%.

The standard WHO resistance tube bioassay found that the mortality rate of the mosquitoes against the various control papers was generally <5%, and in 5–20% in 6 (12.2%) tests. The corrected mortality rates were all below 90% for the five insecticides tested in both the Anhui and Yunnan sites (Fig. 2). According to WHO criteria [4], *An. sinensis* mosquitoes from the two study sites were resistant to pyrethroids, carbamates, organophosphates, and organochlorines. Further, with the exception of malathion, resistance was extremely prevalent, as more than 50% of mosquitoes survived the diagnostic dose for resistance, and in some cases more than 90% of the tested mosquitoes survived the bioassay (Fig. 2). In general, the Anhui population was more resistant than the Yunnan population, as the Anhui population exhibited a significantly lower mortality rate than the Yunnan population for three insecticides tested (permethrin, DDT and malathion) (P<0.01) (Fig. 2).

### Distribution of *kdr* allele and *ace-1* allele frequencies in *An. sinensis* populations

Two types of non-synonymous *kdr* mutation at position 1014 (TTG to TTT and TGT) were observed in the Anhui population. The mutation (TTG → TTT) lead to a change from leucine to phenylalanine (L1014F) and the mutation (TTG → TGT) leads to a leucine to cysteine substitution (L1014C). The L1014F mutation was predominant (70.0–88.9%) and the L1014C mutation (11.1–26.7%) was less common in the Anhui population (Table 1). Wildtype allele frequency was low (<9%). Significantly higher *kdr* mutation frequencies (both L1014F and L1014C alleles) were found in the deltamethrin-resistant mosquitoes than in the susceptible mosquitoes for the Anhui site (Table 1). Except for DDT-susceptible individuals, all genotype frequencies at the *kdr* locus conformed to HWE (P>0.05). Interestingly, no *kdr* mutation was detected in the Yunnan population despite high levels of phenotypic resistance (Table 1). No *kdr* mutation was detected in the lab susceptible strain.

**Table 1:** *kdr* mutation frequency of the study populations and association with resistance to pyrethroid and organochlorine insecticides.

Population	Insecticide	Status	N	Frequency	OR[95% CI]
				L1014F	L1014C	L1014	L1014F	L1014C
Anhui	Deltamethrin	Alive	93	79.0	19.4	1.6	5.44*[1.31,22.54]	5.14*[1.12,23.45]
		Dead	37	72.9	18.9	8.2		
	Permethrin	Alive	57	83.3	12.3	4.4	0.39[0.02,7.08]	0.41[0.02,9.56]
		Dead	15	70.0	26.7	3.3		
	DDT	Alive	67	81.3	12.7	6.0	0.90[0.10,8.15]	0.35[0.03,3.54]
		Dead	9	88.9	11.1	0		
Yunnan	Deltamethrin	Alive	75	0	0	100	-	-
		Dead	26	0	0	100		
	Permethrin	Alive	69	0	0	100	-	-
		Dead	28	0	0	100		
	DDT	Alive	40	0	0	100	-	-
		Dead	61	0	0	100		

Note. The laboratory susceptible strain showed 100% mortality for the three insecticides and no *kdr* mutation was detected. N is the number of individuals tested. L1014F represents a mutated allele from leucine to phenylalanine at codon 1014 of the *para* sodium ion channel gene, L1014C is another mutated allele from leucine to cysteine, and L1014 is the wildtype allele. OR (odds ratio) tests the association between a particular mutation and resistance.

*, P<0.05.

**Figure 3:**
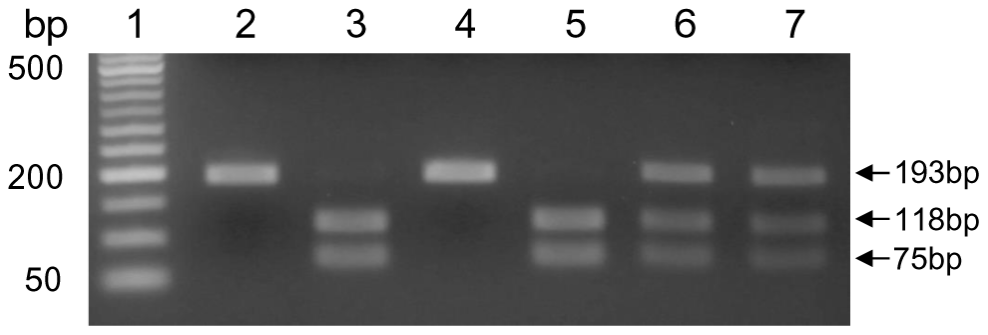
*Anopheles sinensis* G119S mutation detection in *ace-1* by PCR-RFLP assay. Lane 1: 50 bp ladder; lanes 2 and 4: homozygous for *ace-1* wildtype; lanes 3 and 5: homozygous for *ace-1* resistance mutation; lanes 6 and 7: heterozygous for *ace-1* mutation.

To detect the target site mutation in the *ace-1* gene (*ace-1^R^*), a 193-bp fragment was amplified by PCR. The PCR product was digested by *Alu*I restriction enzyme, resulting in 118-bp and 75-bp fragments when a G119S resistant mutation was present (Fig 3). Comparison of PCR-RFLP method with direct sequencing on 40 individuals revealed 100% consistency in *ace-1* mutation detection. The G119S allele was detected in both the Anhui and Yunnan populations, with 58.9% and 38.5% frequencies, respectively. No *ace-1* mutation was detected in the lab susceptible strain. For the Yunnan population, *ace-1* alleles were at HWE, but the Anhui population exhibited a significant heterozygote excess (P<0.05). Significantly higher G119S allele frequency was observed in malathion-resistant mosquitoes than in susceptible mosquitoes in both the Anhui and Yunnan populations (P<0.001), but such a phenomenon was not observed in the bendiocarb-resistant or susceptible populations for both sites (Table 2).

**Table 2:** Mutation frequency of *ace-1* gene in the study populations and association with resistance to organophosphate and carbamate insecticides.

Population	Insecticide	Status	N	*ace-1* genotype			G119S	
				G/G	G/S	S/S	allele frequency	OR[95% CI]
Anhui	Malathion	Alive	97	0	69	28	64.4	3.34*[2.04,5.46]
		Dead	54	19	32	3	35.2	
	Bendiocarb	Alive	83	1	58	24	63.9	0.66[0.32,1.34]
		Dead	24	4	5	15	72.9	
Yunnan	Malathion	Alive	29	0	17	12	70.7	9.56*[4.76,19.20]
		Dead	72	45	25	2	20.1	
	Bendiocarb	Alive	49	16	20	13	46.9	1.77[0.50,6.26]
		Dead	6	2	4	0	33.3	

Note. The laboratory susceptible strain exhibited 100% mortality for the two insecticides and no *ace-1* mutation was detected. N is the number of individuals tested. G119S represents a mutated allele from glycine to serine at the codon of 119 of *ace-1* gene. G/G refers to wildtype genotype, S/S is homozygous mutation, and G/S is heterozygote for the *ace-1* gene. OR (odds ratio) tests the association between a particular mutation and resistance.

*, P<0.05.

### Metabolic enzyme activities and association with resistance

**Figure 4:**
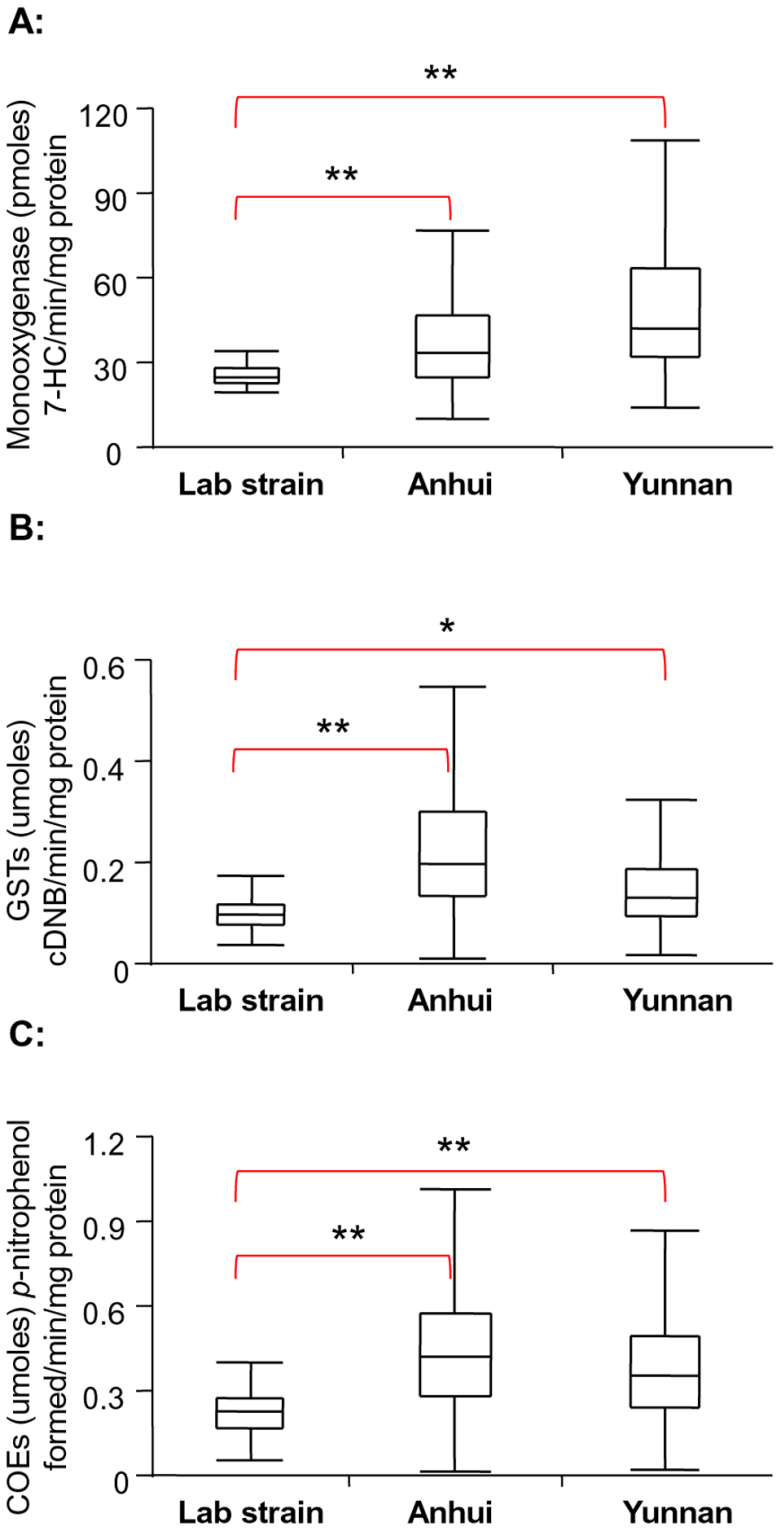
Boxplots of metabolic detoxification enzyme activities in *Anopheles sinensis* populations from laboratory susceptible strain, Anhui and Yunnan populations. The median activity is shown by a horizontal bar; the box denotes the upper and lower quartiles. The vertical lines show the full range of the data set. *, P<0.05 and **, P<0.001 represent variance significantly different from lab strain with pairwise comparison after the analysis of variance. A: P450 monooxygenases; B: glutathione S-transferases; and C: carboxylesterases.

The median P450 activity of the lab strain was 25.3 pmol 7-HC/min/mg protein (ranging from 19.6 to 34.5), the median GST activity was 0.229 µmol cDNB/min/mg protein (ranging from 0.06 to 0.44), and the mean COE activity was 0.104 µmol *p*-nitrophenol formed/min/mg protein (ranging from 0.04 to 0.21). For field-collected mosquitoes, significantly elevated levels of cytochrome P450s, GSTs and COEs were found in the Anhui and Yunnan populations compared with the susceptible lab strain (Fig 4). Overall, F-tests found significantly higher variances in field-collected mosquitoes than in the laboratory strain for both the Anhui and Yunnan populations (F<0.01 for all tests; Fig. 4), suggesting much higher within-population variability in the metabolic detoxification enzyme activities.

**Figure 5:**
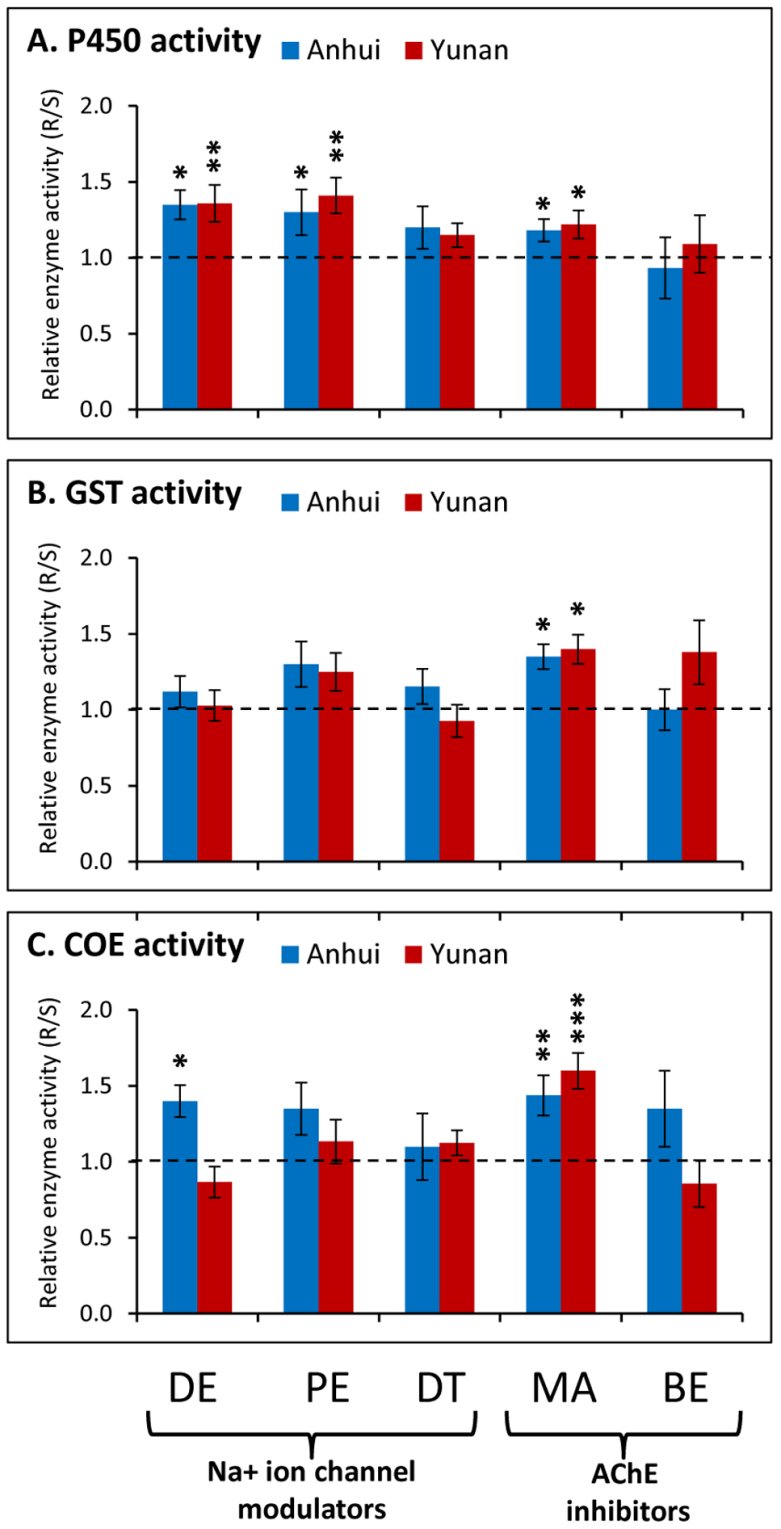
Ratio of metabolic enzyme activities of resistant mosquitoes to susceptible mosquitoes. Resistant individuals were those that survived the standard WHO resistance tube bioassay; susceptible individuals were those that died in the bioassay with the discriminating insecticide dosage. Insecticides and their discriminating dosages tested were: deltamethrin 0.05% (DE), permethrin 0.75% (PE), DDT 4% (DDT), malathion 5% (MA) and bendiocarb 0.1% (BE). A: P450 monooxygenases (P450); B: glutathione S-transferases (GST); and C: carboxylesterases (COE). *, P<0.05; **, P<0.01; and ***, P<0.001.

Comparison of metabolic enzyme activities between the mosquitoes that survived the bioassay (resistant) and those that died in the bioassay (susceptible) would reveal the role of the metabolic enzyme activities in resistance. We found that deltamethrin- permethrin- and malathion-resistant mosquitoes consistently showed significantly higher P450 activities than susceptible mosquitoes (Fig 5A). Further, malathion-resistant mosquitoes showed significantly higher GST activities (Fig. 5B) and COE activities (Fig. 5C) than susceptible mosquitoes, suggesting all three metabolic detoxification enzymes may play a role in malathion resistance.

### Relative importance of target site (*kdr* and *ace-1*) genotypes and metabolic detoxification enzymes in insecticide resistance

**Figure 6:**
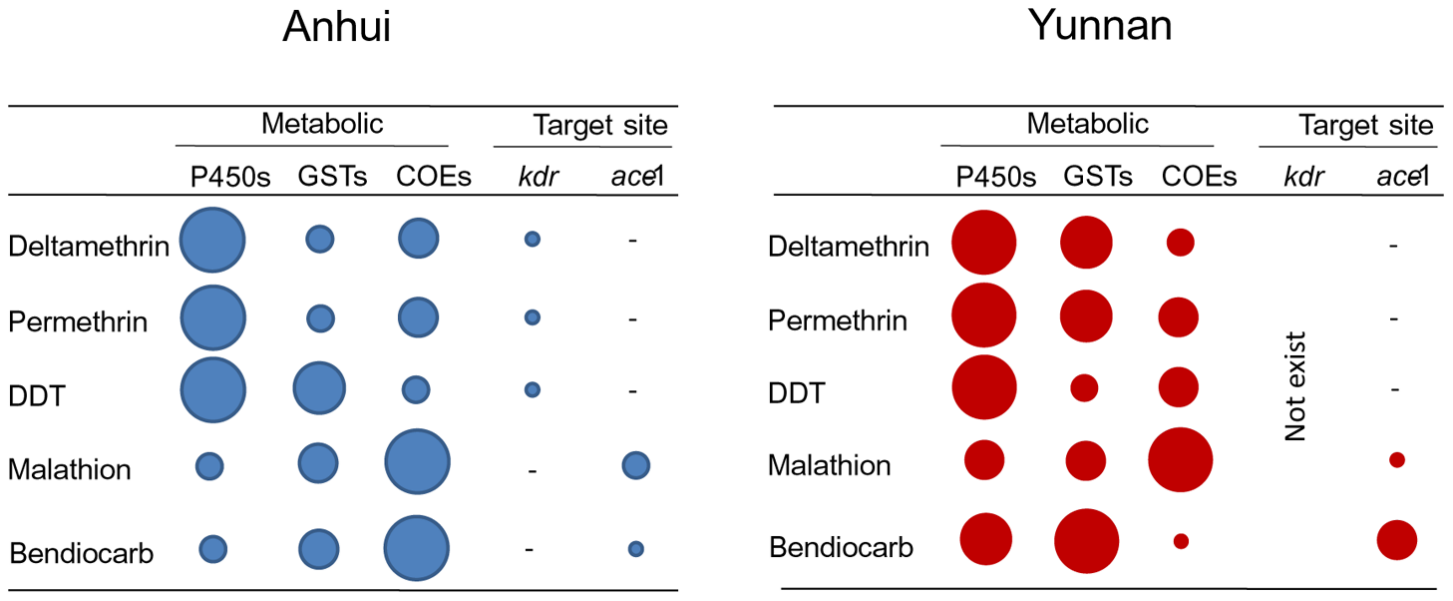
Schematic representation of the relative importance of target-site insensitivity and metabolic detoxification enzymes in resistance to multiple classes of insecticides in two populations of *Anopheles sinensis*, Anhui and Yunnan. Circle size reflects the relative impact of the mechanism. Variable importance for a particular predictor is the sum, across all nodes, of the improvement scores the predictor has when it acts as a splitter. The most important variable is always expressed as 100%. P450s: P450 monooxygenases; GSTs: glutathione S-transferases; COEs: carboxylesterases; *kdr*: knockdown resistance; *ace-1*: acetylcholinesterase gene 1; and “-”: genotype not examined.

One unsolved key question in insecticide resistance research is the determination of the relative contributions of target site insensitivity and various metabolic detoxification enzymes to resistance. To answer this question, we conducted a CART analysis that simultaneously took *kdr* and *ace-1* genotypes and three metabolic enzyme activities into consideration. Because resistance to malathion and bendiocarb involves the same target site (*ace-1*) and resistance to deltamethrin, permethrin and DDT involves a different target site (*kdr*), we analyzed resistance to malathion and bendiocarb separately from the other three insecticides. The CART analysis revealed that for resistance to deltamethrin, permethrin, and DDT, the most important variable was P450 activity, followed by GST or COE activity, and *kdr* mutation played a small role (Fig 6; Table S1). For resistance to malathion, the most important variable was COE activity, followed by GST and P450 activity, and G119S mutation was less important. These were consistent for the Yunnan and Anhui populations (Fig. 6). The role of COEs in resistance to bendiocarb varied between the Yunnan and Anhui populations, and the important role of GSTs was consistent. Overall, these results suggest metabolic resistance was the most important resistance mechanism for the four classes of insecticides tested.

### Insecticide use survey and residual chemical analysis

Overall, rice is the major crop in the two study sites. Other crops include wheat, corn, sugarcane, banana, and vegetables. Pyrethroids, organochlorine, organophosphates and carbamates were all used in agricultural pest control (Table S2). The organophosphate-based insecticides were more commonly used than pyrethroids. The common insecticides used for agricultural pest control included lambda-cyhalothrin, beta-cypermethrin, dichlorvos, chlorpyrifos, malathion and propoxur. The insecticides used for indoor residual spraying and bed net treatment included dimefluthrin, alpha-cypermethrin, meperfluthrin and D-prallethrin. Although the insecticide usage surveys were conducted in limited number of houses (n = 20 per site), the survey results showed diverse types of insecticides being used in the study sites. Residual insecticide analysis in samples from the Anhui study site detected chlorpyrifos in soil samples with concentration ranging from 21–130 ppb, but no chlorpyrifos was detected in the water samples (Table S3). Deltamethrin was not detected in either the soil or water samples. The analytes of interest (deltamethrin and chlorpyrifos) were all detected in the positive control water and soil samples, confirming the soundness of the analytic technique.

## Discussion

The study demonstrated that field populations of *An. sinensis* from central China (Anhui) and southern China (Yunnan) developed high resistance to four classes of insecticides tested, including pyrethroid (deltamethrin and permethrin), organochlorine (DDT), organophosphate (malathion) and carbamate (bendiocarb). The *An. sinensis* population from Anhui was more resistant to permethrin, DDT, and malathion than the population from Yunnan, while resistance levels to deltamethrin and bendiocarb were similar. Interestingly, among the 300 samples from Yunnan tested for *kdr* mutation, no *kdr* mutation was detected. Therefore, the target site *kdr* mutation at L1014 is not the resistance mechanism to pyrethroids and DDT in the Yunnan population. On the other hand, *kdr* mutation reached near fixation for the Anhui population, and our CART analysis suggests that *kdr* mutation only played a small role in pyrethroids and DDT resistance. Similarly, target site mutation at the *ace-1* gene makes a small contribution to resistance to organophosphate and carbamate. This is consistent for both the Anhui and Yunnan populations. Therefore, we conclude that under long-term high insecticide selection pressure, mosquitoes have evolved a strong metabolic resistance to various classes of insecticides.

Because the same bioassayed mosquitoes in our study were tested for *kdr* and *ace-1* mutations and three detoxification enzyme activities, using the CART statistical method, we were able to statistically determine the importance of these individual factors in resistance to multiple insecticides. This analysis found that *kdr* and *ace-1* mutations played a small role in resistance to the four classes of insecticides tested, P450 activity was the most important mechanism in mosquito resistance to pyrethroids and organochlorines and COE monooxygenases was most important to organophosphate and carbamate resistance. When a mosquito population lacked *kdr* mutation (as in the Yunnan population), GSTs played a large role in pyrethroid resistance in comparison to the Anhui population, in which *kdr* mutation was very prevalent. Previous investigations on metabolic activity in African *An. gambiae* confirmed that monooxygenases played an important role in the resistance to pyrethroids while COE was the major metabolic mechanism for organophosphates [47]–[49]. This correlates with our findings. Several studies have found correlation between L1014F allele frequency and resistance to pyrethroids in *An. gambiae* populations from West Africa [48], [50]–[53]. Thus, it is important to experimentally verify the results from the CART analysis that only statistically teased out the specific contribution of each resistance mechanism. For example, the metabolic enzymes may be inhibited using various synergists prior to insecticide resistance bioassay, such as synergists PBO (4% pyperonyl butoxide), DEF (0.25% S.S.S-tributyl phosphotritioate), DEM (8% diethyl maleate) and TPP (10% Triphenyl phosphate) which are known inhibitors of multi-function oxidases (MFOs), glutathione S-transferases (GSTs), non-specific esterases (NSEs) and carboxylesterase(COEs), respectively [54]–[58]. Through comparison of mosquitoes pre-exposed to synergists with the appropriate controls, the effects of specific metabolic enzymes on resistance can be determined.

The lack of *kdr* mutation in *An. sinensis* population in Yunnan Province was not unique to our particular study site (Lianghe and Yingjiang counties). We have previously examined *An. sinensis* from Mengla and Yuanyang counties of Yunnan Province and did not detect any *kdr* mutation in 186 samples [23], despite the fact that these mosquito populations have been, and are currently, experiencing strong insecticide selection pressures. Low *kdr* mutation frequency (<30%) was reported in an *An. sinensis* population from another southern China province, Guangxi [59]. The high *kdr* mutation frequency observed in the central China Anhui study site was consistent with other studies on *An. sinensis* throughout central China. For example, we detected >90% *kdr* mutation frequency in Hunan, Hubei and Jiangsu Provinces, China [23]. Tan *et al* [60] reported a 95–100% *kdr* mutation frequency in an *An. sinensis* population from Jiangsu Province in central China. In addition to the predominant L1014F *kdr* allele, we found L1014C allele with considerable frequency. Further, we detected a significant positive association between L1014C mutation and resistance to deltamethrin, but not to DDT and permethrin resistance. The role of L1014C mutation on insecticide resistance should be further investigated by increasing the number of sample sites. It is not clear what caused the lack of *kdr* mutation in the Yunnan population. One interesting possibility is that the lack of gene flow between central China and the mountainous Yunnan Province prevents *kdr* mutation from being spread to the population in Yunnan. We are currently examining the *An. sinensis* population genetic structure in China to determine the role of gene flow on the spread of *kdr* mutation.

The high level and multiple insecticide resistance of *An. sinensis* in China may result from prolonged and extensive use of insecticide for agricultural pest control and public health disease vector control. The detected insecticide residues of organophosphates (chlorpyrifos) in soil in Anhui site further suggest that insecticide residues in the larval environment of mosquitoes through agricultural pest control spray may be an important factor that selected for insecticide resistance. It is possible selection pressure from larval environment may be very strong as mosquito larvae are confined to the aquatic habitats with residual insecticides and constantly exposed to insecticides in the aquatic habitats. Adult mosquitoes are mobile and may exhibit behavioral avoidance to the insecticide [61], [62]. Whether selection pressure from larval exposure to insecticides favors metabolic resistance more than mutational target site resistance is an interesting question for future research.

Resistance to multiple classes of insecticides is becoming a common problem in various disease vector species. Reported multiple resistance in mosquito vectors includes *An. gambiae* [47]-[49], [63], *An. arabiensis* [64], *An. funestus* [65], *Culex quinquefasciatus* [47], *Aedes aegypti* and *Ae. albopictus* [66] in Africa, and *An. culicifacies, An. subpictus, An. nigerrimus, An. peditaeniatus* [56] and *Cx. quinquefasciatus* [67], [68] in Asia, and *Ae. Aegypti* in South America [69]. Multiple insecticide resistance impedes the current front-line vector-borne disease control programs, which are primarily based on the use of pyrethroids. The present study suggests that long-term use of various classes of insecticides, in rotation or combination, will eventually select vector populations resistant to the major classes of insecticides. Our results have important implications for *Anopheles* and other mosquito vector control strategies. Organophosphate and carbamate insecticides may have limited applications in disease vector control, and rotation or combinational use with these insecticides may not be effective as the mosquito populations are already highly resistant. Adding appropriate synergists to the IRS formulation may help improve the effectiveness of the insecticides. Developing and implementing alternative efficient vector control methods that are not reliant on pyrethroids, organophosphate and carbamates—such as home improvement [70], odor-baited traps [71], larval resource reduction [72] and biological control [73], microbial insecticides and new classes of insecticides — presents an urgent challenge.

## Supporting Information

Table S1 Relative importance value of variables used in the classification and regression trees (CART) analysis. Variable importance, for a particular predictor, is the sum, across all nodes, of the improvement scores that a predictor has when it acts as a splitter. The most important variable is expressed as 100%.
(DOCX)

Table S2 Survey of insecticide usage in Anhui and Yunan study sites in China for agricultural pest and public health vector control.
(DOC)

Table S3 Analysis of insecticide residues in water and soil samples in the Anhui study site.
(DOC)
