# Correction: Multiple Resistances and Complex Mechanisms of *Anopheles sinensis* Mosquito: A Major Obstacle to Mosquito-Borne Diseases Control and Elimination in China

**DOI:** 10.1371/journal.pntd.0002967

**Published:** 2014-06-12

**Authors:** The PLOS Neglected Tropical Diseases Staff

In the Funding section, one of the grant numbers from the funder the National Institutes of Health is listed incorrectly. The correct grant number is: U19 AI089672.
