# Supplementary material for: Multiple Resistances and Complex Mechanisms of Anopheles sinensis Mosquito: A Major Obstacle to Mosquito-Borne Diseases Control and Elimination in China
Source: PLoS Negl Trop Dis. 2014 May 22;8(5):e2889. doi: 10.1371/journal.pntd.0002889 (PMC4031067; doi:10.1371/journal.pntd.0002889)
Supplement: Table S1 — Relative importance value of variables used in the classification and regression trees (CART) analysis. Variable importance, for a particular predictor, is the sum, across all nodes, of the improvement scores that a predictor has when it acts as a splitter. The most important variable is expressed as 100%. (DOCX) [file pntd.0002889.s001.docx]

**Table S1** Relative importance value of variables used in the classification and regression trees (CART) analysis. Variable importance, for a particular predictor, is the sum, across all nodes, of the improvement scores that a predictor has when it acts as a splitter. The most important variable is expressed as 100%.

| **Population** | **Insecticide** | **Metabolic enzymes*** | | | **Target site mutation** | |
| --- | --- | --- | --- | --- | --- | --- |
|  |  | P450 | GST | COE | *kdr* | *ace-1* |
| **Anhui** | Deltamethrin | 100 | 5.1 | 32.3 | 19.4 | 0 |
|  | Permethrin | 100 | 48.9 | 67.6 | 11.7 | 0 |
|  | DDT | 100 | 75.4 | 33.9 | 9.5 | 0 |
|  | Malathion | 48 | 53.9 | 100 | 15 | 34.7 |
|  | Bendiocarb | 34.9 | 68.8 | 100 | 0 | 2.7 |
|  |  |  |  |  |  |  |
| **Yunnan** | Deltamethrin | 100 | 81.2 | 26.4 | 0 | 0 |
|  | Permethrin | 100 | 79.7 | 62.6 | 0 | 0 |
|  | DDT | 100 | 48 | 73.9 | 0 | 0 |
|  | Malathion | 70 | 72.3 | 100 | 0 | 24.1 |
|  | Bendiocarb | 88.4 | 100 | 24.4 | 0 | 56.6 |

* P450 stands for P450 monooxygenases, GST is glutathione s-transferases, and COE is carboxylesterases.
