# Supplementary material for: Multiple Resistances and Complex Mechanisms of Anopheles sinensis Mosquito: A Major Obstacle to Mosquito-Borne Diseases Control and Elimination in China
Source: PLoS Negl Trop Dis. 2014 May 22;8(5):e2889. doi: 10.1371/journal.pntd.0002889 (PMC4031067; doi:10.1371/journal.pntd.0002889)
Supplement: Table S2 — Survey of insecticide usage in Anhui and Yunan study sites in China for agricultural pest and public health vector control. (DOC) [file pntd.0002889.s002.doc]

**Table S2** Survey of insecticide usage in Anhui and Yunan study sites in China for agricultural pest and public health vector control.

| **Study site** | **Crops planted** | **Insecticide class** | **Agricultural pest control** | **Public health vector control for human and animal protection** |
| --- | --- | --- | --- | --- |
| Anhui | Rice, wheat, corn | Pyrethroid | Beta-cypermethrin, Lambda-cyhalothrin | Deltamethrin, Alpha-Cypermethrin, Dimefluthrin, Deperfluthrin, [D-Prallethrin](https://www.google.com/url?sa=t&rct=j&q=&esrc=s&source=web&cd=7&cad=rja&ved=0CF4QFjAG&url=http%3A%2F%2Fwww.agropages.com%2Fagrodata%2FDetail-3611.htm&ei=ghCdUa_HNMi9igKQo4GwAg&usg=AFQjCNGfoAPgYDRrxWthQ7myQTMrZooccw&bvm=bv.46751780,d.cGE) |
|  |  | Organochlorine | Dichlorovos |  |
|  |  | Organophosphate | Chlorpyrifos, Malathion |  |
|  |  | Carbamate | Aminocarb, Propoxur |  |
|  |  |  |  |  |
| Yunnan | Rice, banana, corn, sugarcane | Pyrethroid | Meothrin, Fenpropathrin, Lambda-Cyhalothrin | Deltamethrin, Alpha-Cypermethrin, Dimefluthrin, Alpha-Meperfluthrin, [D-Prallethrin](https://www.google.com/url?sa=t&rct=j&q=&esrc=s&source=web&cd=7&cad=rja&ved=0CF4QFjAG&url=http%3A%2F%2Fwww.agropages.com%2Fagrodata%2FDetail-3611.htm&ei=ghCdUa_HNMi9igKQo4GwAg&usg=AFQjCNGfoAPgYDRrxWthQ7myQTMrZooccw&bvm=bv.46751780,d.cGE) |
|  |  | Organochlorine | Dichlorovos |  |
|  |  | Organophosphate | Chlorpyrifos, Phoxim |  |
|  |  | Carbamate | Propamocarb |  |
