# Supplementary material for: Multiple Resistances and Complex Mechanisms of Anopheles sinensis Mosquito: A Major Obstacle to Mosquito-Borne Diseases Control and Elimination in China
Source: PLoS Negl Trop Dis. 2014 May 22;8(5):e2889. doi: 10.1371/journal.pntd.0002889 (PMC4031067; doi:10.1371/journal.pntd.0002889)
Supplement: Table S3 — Analysis of insecticide residues in water and soil samples in the Anhui study site. (DOC) [file pntd.0002889.s003.doc]

**Table S3** Analysis of insecticide residues in water and soil samples in the Anhui study site.

|  |  |  | **Detected concentration (ppb)** | |
| --- | --- | --- | --- | --- |
| **Sample** | **GPS coordinates** | **Sample type** | **Deltamethrin** | **Chlorpyrifos** |
| 1 (Positive control) |  | Water | 10 | 70 |
|  |  | Soil | 580 | 300 |
| 2 (Negative control) | N33.2311, E117.8671 | Water | <LOD | <LOD |
|  |  | Soil | <LOD | <LOD |
| 3 | N33.2284, E117.8488 | Water | <LOD | <LOD |
|  |  | Soil | <LOD | 130 |
| 4 | N33.2260, E117.8459 | Water | <LOD | <LOD |
|  |  | Soil | <LOD | 26 |
| 5 | N33.1582, E117.8140 | Water | <LOD | <LOD |
|  |  | Soil | <LOD | 44 |
| 6 | N33.1581, E117.8161 | Water | <LOD | <LOD |
|  |  | Soil | <LOD | 21 |

Note. LOD stands for the limit of detection (0.2 ppb and 2 ppb for deltamethrin and chlorpyrifos, respectively).
